# ER-transiting bacterial toxins amplify STING innate immune responses and elicit ER stress

**DOI:** 10.1128/iai.00300-24

**Published:** 2024-07-26

**Authors:** Catherine Schlenker, Katharina Richard, Sofia Skobelkina, R. Paige Mathena, Darren J. Perkins

**Affiliations:** 1 Program in Oncology University of Maryland, Baltimore (UMB), School of Medicine, Baltimore, Maryland, USA; 2 Department of Microbiology and Immunology, School of Medicine, Baltimore, Maryland, USA; Stanford University School of Medicine, Stanford, California, USA

**Keywords:** STING, cGAS, pertussis toxin, cholera toxin, type I interferon, ADP ribosylation, GPCR, endoplasmic reticulum

## Abstract

The cGAS/STING sensor system drives innate immune responses to intracellular microbial double-stranded DNA (dsDNA) and bacterial cyclic nucleotide second messengers (e.g., c-di-AMP). STING-dependent cell-intrinsic responses can increase resistance to microbial infection and speed pathogen clearance. Correspondingly, STING activation and signaling are known to be targeted for suppression by effectors from several bacterial pathogens. Whether STING responses are also positively regulated through sensing of specific bacterial effectors is less clear. We find that STING activation through dsDNA, by its canonical ligand 2′-3′ cGAMP, or the small molecule DMXAA is potentiated following intracellular delivery of the AB_5_ toxin family member pertussis toxin from *Bordetella pertussis* or the B subunit of cholera toxin from *Vibrio cholerae*. Entry of pertussis toxin or cholera toxin B into mouse macrophages triggers markers of endoplasmic reticulum (ER) stress and enhances ligand-dependent STING responses at the level of STING receptor activation in a manner that is independent of toxin enzymatic activity. Our results provide an example in which STING responses integrate information about the presence of relevant ER-transiting bacterial toxins into the innate inflammatory response and may help to explain the *in vivo* adjuvant effects of catalytically inactive toxins.

## INTRODUCTION

In mammalian cells, an overlapping network of innate immune sensors detects molecular patterns associated with microbes. These molecular patterns can include bacterial structural elements as well as their secreted effectors and metabolic products (1). Within this sensor network, the cGAS/STING pathway is a two-component system that is well described as a detector of microbial double-stranded DNA (dsDNA) and bacterially produced cyclic nucleotides. In the presence of cytosolic dsDNA, the enzymatic activity of the cyclic GMP-AMP synthase (cGAS) is triggered and synthesizes the cyclic nucleotide 2′-3′ cyclic GMP-AMP (cGAMP) (2, 3). cGAMP then functions as a second messenger that binds to the endoplasmic reticulum (ER)-localized Stimulator of Interferon Genes (STING, also referred to as MITA and MPYS) (4 – 6). In addition to cGAMP endogenously generated by cGAS, STING can be directly activated by select bacterial cyclic second messengers including c-di-AMP released into the cytosol (7) providing STING with a proximate role in sensing the presence of intracellular bacteria.

Once activated by cyclic dinucleotides of endogenous or exogenous origin on the ER, STING dimers form and become stabilized by a disulfide linkage (8), and through subsequent steps still being elucidated, STING translocates from the ER ultimately to an ER-Golgi intermediate compartment (ERGIC) (9 – 11). Translocated STING functions as an organizing signaling scaffold to drive a transcriptional program mediated by the transcription factors IRF3 and NF-κB, producing a robust type I interferon response. STING activation also simultaneously drives cell-intrinsic defense through triggering the autophagic cascade, and autophagy is critical for the STING-dependent restriction of some microbial infections (12).

The importance of the cGAS/STING system in responding to microbes is underscored by the fact that cGAS and STING are inhibited by effectors from several viral and bacterial pathogens. Notably, the *Shigella* effector, IPAJ, blocks the rate-limiting translocation of STING from the ER to the ERGIC, inhibiting type I interferon transcriptional responses (9), and multiple Pox virus encodes cGAMP-specific nucleases, which can prevent cGAS-dependent STING activation (13).

To date, no instances in which STING has a positive role in sensing and initiating an innate response to bacterial toxins/effectors have been described; however, there are several examples in which bacterial toxins and secreted effectors are either sensed directly by physical interaction or sensed through their homeostasis perturbing enzymatic activities as molecular patterns thereby triggering the activation of pattern recognition receptors (14 – 16).

The A/B_5_ family of bacterial toxins share a structural architecture with the non-enzymatic B subunits directing the entry and transit of the enzymatic toxic A subunit. The pertussis toxin (PTx) produced by the human pathogen *Bordetella pertussis,* the causative agent of whooping cough disease, is a classic example of an AB_5_ toxin. PTx is a multi-subunit (A/B_5_) protein toxin that is secreted into the lungs during *B. pertussis* infection and is required for virulence of *B. pertussis* (17). PTx enters by endocytosis into the cytosol of both immune and non-immune cells and subsequently transits through the ER to release the catalytically active A subunit back into the cytosol. Functionally, PTx encodes one known enzymatic activity. PTx ADP ribosylates G_α_i type G protein complexes, uncoupling these signaling complexes from their cognate G protein-coupled receptors (GPCRs). Such perturbation of GPCR signaling impacts many cellular functions, and PTx is known to have immune-modulatory capabilities including as an adjuvant. However, despite the fact that PTx has long been known to traffic intracellularly, interactions between PTx and intracellular innate immune receptors are undescribed (18). The cholera toxin (CTx) shares AB_5_ structural organization with PTx and similarly transits through the ER as a requirement to activate the catalytic subunit of the toxin. Cholera toxin is also capable of functioning as a mucosal adjuvant by undefined mechanisms. However, the impact, if any, of ER transit by the PTx and CTx bacterial toxins on innate immune responses by the host cell is unknown.

We have investigated whether the intracellular cGAS/STING sensing pathway is sensitive to the presence of intracellular PTx or CTx. We have found that exposure of macrophages to PTx or CTx B subunit leads to stronger ligand-dependent STING-dependent responses in a manner independent of the catalytic activity of the toxins. These data are the first example in which ER resident STING responses may be positively regulated by the presence of some bacterial toxins.

## MATERIALS AND METHODS

### qRT-PCR

Total mRNA was isolated from cells using TRIPure (Sigma/Roche Cat No. 11667165001) reagent, according to the manufacturer’s instructions. A total of 1 µg RNA was used in cDNA synthesis using the iScript cDNA synthesis kit (BioRad) according to the manufacturer’s instructions. Quantitative real time PCR (qRT-PCR) was performed on cDNA using an Applied Biosystems Quant Studio 3 system with the Power SYBR Green reagent (Applied Biosystems) and transcript-specific primers as previously described (19). Levels of mRNA for specific genes are reported as relative gene expression normalized to that of untreated cells [“fold induction” (20)]. The housekeeping gene encoding glyceraldehyde 3 phosphate dehydrogenase (GAPDH) was used for the normalization of RNA levels within each sample. In addition to previously published primer sequences for the detection of cytokine gene expression, the following primer sets were used to detect mRNA: (GAPDH FWD- AGC CTC GTC CCG TAG ACA AAAT; REV- TGG CAA CAA TCT CCA CTT TGC) (IFN-b FWD- CAC TTG AAG AGC TAT TAC TGG AGG G; REV- CTC GGA CCA CCA TCC AGG) (IP10 FWD- CCA CGT GTT GAG ATC ATT GCC; REV- GCC CTT TTA GAC CTT TTT TGG C)

### Cell lines and mice

Primary bone marrow-derived macrophages (BMDMs) were prepared, as described previously (21). Briefly, bone marrow was harvested from C57BL6/J mice and cultured in a standard medium supplemented with a 20%–25% LADMAC cell-conditioned medium (22). After 48 hrs, non-adherent cells were removed, and adherent cells were cultured for an additional 7 days. Adherent cells were harvested using 0.25% Trypsin-EDTA (Life Technologies, Grand Island, NY) and replated for experimental use. The RAW264.7 cell line (purchased from Cyagen) was maintained in Dulbecco’s modified Eagles medium (DMEM) supplemented with Penn/Strep, L-glutamine, and 10% fetal bovine serum (FBS). The immortalized intestinal epithelial line MODE K (23) was a kind gift from Dr. Dana Philpott (University of Toronto).

### Antibodies and reagents

Antibodies directed against IRF3 (clone D83B9), P-IRF3 (clone 4D4G), STING (clone D1V5L), P-STING ser 366 (clone D8F4W), P-TBK-1 ser 172 (clone D52C2), P-STAT1 Y701 (clone 58DG), p65 (clone D14E12), P-p65 ser 536 (clone 93H1), P-p38 (clone D3F9), LC3 A/B (clone D3U4C), Bip (clone C50B12), protein disulfide isomerase (PDI) (clone C81H6), COX2 (clone D5H5), and β-tubulin (clone 9F3) were purchased from Cell Signaling (Danvers, MA). Anti-viperin (clone MaP.VIP) was purchased from Millipore. 2′-3′-cGAMP was purchased from Tocris (cat no.5945). Interferon stimulatory DNA (ISD-DNA) was purchased from Invivogen (cat no. tlrl-isdn). Sodium tauroursodeoxycholate (TUDC) was purchased from Selleckchem (cat no. S7896). Lipofectamine 2000 transfection reagent was purchased from Invitrogen (cat no. 11668-027). Purified WT PTx was purchased from Tocris (cat no. 3097). R9K;E129A mutant pertussis toxin was purchased from List Labs (cat no. 184). Purified cholera toxin was purchased from List Labs (cat no. 100B). Cholera toxin B expressed in HEK293T mammalian cells was purchased from Sigma (cat no. SAE0069). Complete mini and PhosSTOP inhibitor cocktail tablets were purchased from Roche (cat no. 11836170001 and 4906845001).

### Stimulation of bone marrow-derived macrophages and RAW264.7 cells

For STING activation experiments involving pertussis toxin, BMDMs were plated at a density of 2 × 10^5^ cells per well in 12-well plates and allowed to adhere overnight. On the following day, cell culture media were changed and purified with WT or R9K;E129A pertussis toxin, resuspended in H_2_0, added to individual wells at a concentration of 250 ng/mL, and allowed to intoxicate overnight (16–18 hrs). The next morning, cell culture media were again replaced with fresh media, and BMDMs were immediately stimulated with 5,6-dimethyl-9-oxo-9*H*-xanthene-4-acetic acid (DMXAA) (10 µg/mL for the indicated time) (Tocris cat. no. 5601/10), resuspended in dimethyl sulfoxide (DMSO) (Sigma) or 2′-3′-cGAMP (Tocris cat no. 5945), and resuspended in water for indicated times. For BMDM experiments involving cholera toxin intoxication, BMDMs were plated similarly but intoxicated for 3 hrs prior to stimulation. For cholera toxin B treatment experiments, cells were stimulated overnight (25–100 ng/mL). Following stimulation, total RNA was harvested from cells with the TRIpure reagent (for mRNA expression analysis), or whole-cell lysates were harvested using BI lysis buffer (for western blot) (20 mM HEPES, 1.0% Triton X-100, 0.1% SDS, 150 mM NaCl, Complete mini tablet, and PhosSTOP tablet). For all PTx or CTx B experiments, we found that subconfluent cell density and fresh tissue culture media were important to achieve experiment-to-experiment consistency in STING responses.

### dsDNA transfection of RAW264 cells

RAW264.7 cells were plated at a density of 2.2 × 10^5^ cells per well in a 12-well plate and allowed to adhere for at least 3 hrs overnight. Individual wells of adhered RAW264.7 cells were then intoxicated by the addition of PTx, or vehicle, overnight (16–18 hrs). The following morning, cell culture media were removed and replaced with 500 µL of fresh culture media. Individual wells were transfected with 10 µg of ISD-DNA oligo (Invivogen) using Lipofectamine 2000 according to the manufacturer’s instructions for times indicated in individual experiments.

### Quantitation of secreted cytokines

Cytokine/chemokine levels in macrophage culture supernatants were analyzed by enzyme linked immunosorbent assay (ELISA) at the Cytokine Core Laboratory (University of Maryland, School of Medicine).

### Immunoblot analysis

Immunoblotting was carried out substantially as previously described (24). Whole-cell lysates from macrophage cultures were obtained by the addition of BI lysis buffer (20 mM HEPES, pH 6.8, 1.0% Triton X-100, 0.1% SDS, 150 mM NaCl, and Complete mini and PhosSTOP tablets) and subsequent incubation at 4°C. Cell lysates were separated by denaturing SDS-PAGE and subsequent transfer to polyvinylidene fluoride (PVDF) membranes. Blots were incubated overnight in relevant primary antibodies (identified above) at 4°C, were washed three times with phosphate buffered saline (PBS), and then were incubated with the appropriate horseradish peroxidase (HRP)-conjugated secondary antibody [Jackson HRP anti-rabbit (catalog number 111-035-003) or Jackson HRP anti-mouse (catalog number 115-035-003i), Jackson Immuno-chemicals]. Blots were developed following incubation in the ECL Plus Western Blotting Detection Reagent (Amersham Bioscience).

### Statistical analysis

Statistical analysis, when applied, was done using the Prism software v5.0.

All *t*-tests were done using a two-tailed analysis.

## RESULTS

**Fig 1:**
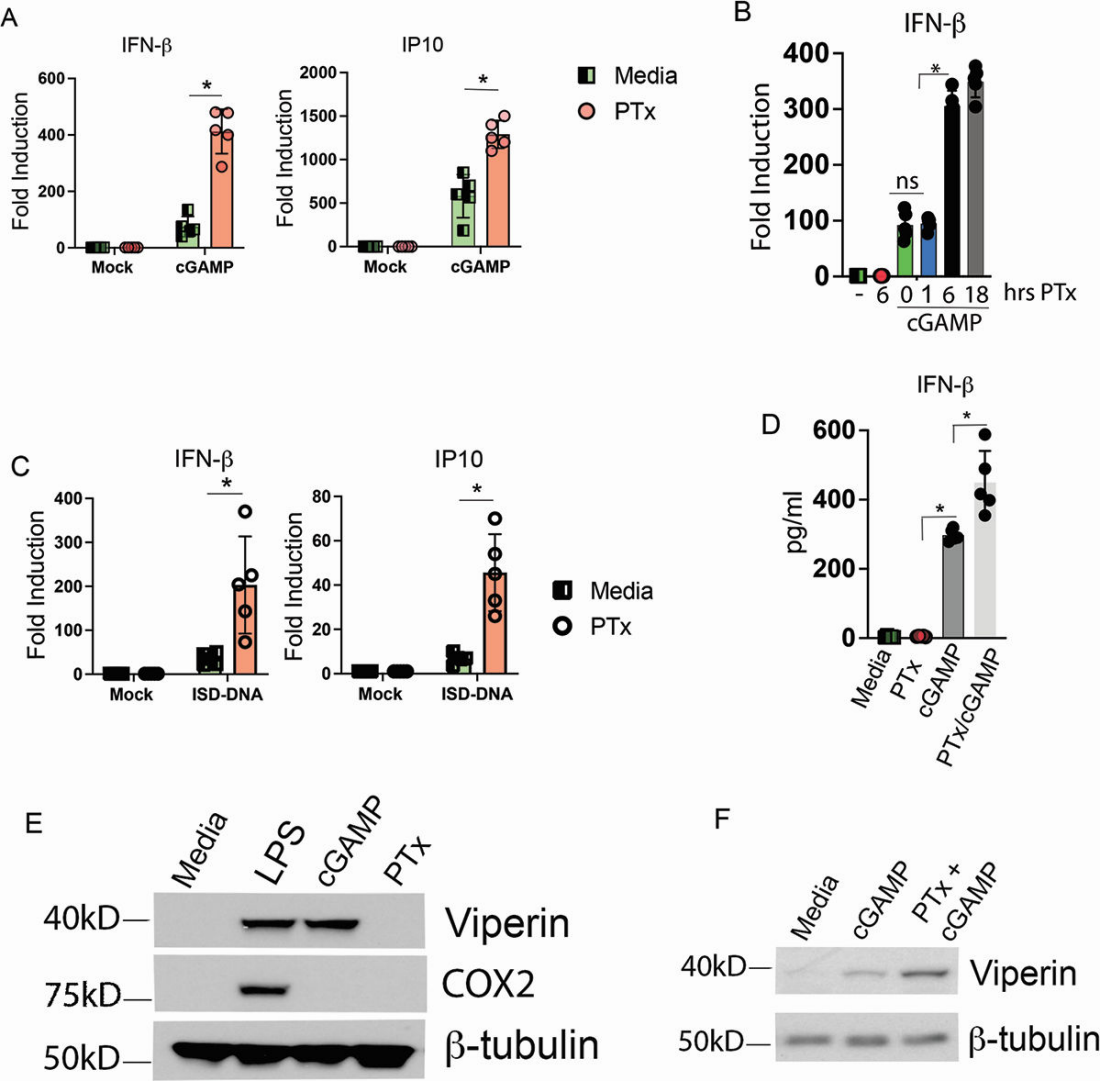
Pertussis toxin intoxication amplifies STING-dependent innate immune responses to cyclic nucleotides and dsDNA. (**A**) Primary murine BMDMs were cultured in media alone or intoxicated overnight with 250 ng/mL of purified PTx. The following morning, media containing toxin were removed and replaced with fresh culture media. BMDMs were subsequently stimulated by the addition of 10 µg/mL cGAMP to culture media for 4 hrs. Total RNA was harvested, and the expression of indicated genes was quantified by qRT-PCR. ^*^ *P* = 0.00234 left panel; ^*^ *P* = 0.0115 right panel. (**B**) Primary BMDMs were treated with 250 ng/mL PTx alone for 6 hrs or pre-treated with 250 ng/mL of PTx for 1, 6, or 18 hrs followed by changing of culture media and immediate 4-hr treatment with 10 µg/mL cGAMP. Total RNA was harvested, and the expression of indicated genes was quantified by qRT-PCR. ^*^ *P* = 0.001. (**C**) RAW264.7 cells were intoxicated overnight with 250 ng/mL of purified pertussis toxin. The following morning, media containing toxin were changed, and cells were transfected with 5 µg total ISD dsDNA for 5 hrs. Total RNA was harvested, and the expression of indicated genes was quantified by qRT-PCR. ^*^ *P* = 0.00056 left panel; ^*^ *P* = 0.00033 right panel. *n* = 5. (**D**) Cell culture supernatants harvested at 4 hrs from macrophages treated as in (**A**) were analyzed for the expression of IFN-β protein by ELISA. ^*^ *P* = 0.000345 for cGAMP; ^*^ *P* = 0.0045 for PTx/cGAMP. (**E**) BMDMs were treated for 18 hrs with media alone, 10 ng/mL *Escherichia coli* lipopolysaccharide (LPS), 10 µg/mL cGAMP, or 250 ng/mL of purified pertussis toxin. Cells were lysed, and lysates were probed using antibodies against the indicated proteins by western blot. (**F**) BMDMs were treated for 18 hrs with media alone or 250 ng/mL of purified pertussis toxin and subsequently stimulated for 8 hrs with 10 µg/mL cGAMP.

To determine whether the intoxication of macrophages by PTx impacts STING-dependent innate immune responses, we treated BMDMs with purified PTx (250 ng/mL) (a dose derived from the experimental design in PTx literature utilizing an *in vitro* cell culture (25 – 27). As the ADP ribosylation of G_α_i proteins by PTx is known to be durable for over 24 hrs (25, 26), we initially tested a 16–18-hr incubation time of the toxin to ensure maximal effect. Following overnight intoxication with PTx, culture media containing toxin were replaced with fresh media, and STING was activated by the addition of cGAMP to the culture medium for an additional 4 hrs. cGAMP-dependent activation of STING was assayed by quantifying the expression of STING-dependent cytokine genes *Ifnb1* (encoding IFN-β) and *Cxcl10* (encoding IP10) using qRT-PCR. No induction of either IFN-β or IP10 was observed with PTx treatment alone (Fig. 1A); however, intoxication of BMDMs by PTx resulted in a significant increase in cGAMP-dependent *Ifnb1* and *Cxcl10* expression when compared to non-intoxicated BMDMs (Fig. 1A). To determine whether prolonged intoxication by PTx was required to potentiate STING cytokine induction, we pre-treated BMDMs with PTx for 0, 1, 6, or 18 hrs followed by 4 hrs of cGAMP stimulation. One hour of Ptx pre-treatment did not impact STING responses, and at least 6 hrs of pre-exposure was required to increase cGAMP-dependent *Ifnb1* transcription (Fig. 1B). Similarly to our previous experiment (Fig. 1A), no induction of either IFN-β or IP10 transcript was observed following 6 hrs of PTx treatment alone (Fig. 1B).

To determine whether PTx intoxication could also increase STING-dependent responses triggered by cytosolic dsDNA, which generates cGAMP through cGAS activation, RAW264.7 cells were intoxicated, or not, for 16–18 hrs with PTx (250 ng/mL) and were transfected with dsDNA oligos (ISD-DNA) for 5 hrs, and transcription of *Ifnb1* and *Cxcl10* was quantified by qRT-PCR. PTx treatment significantly increased both *Ifnb1* and *Cxcl10* transcription in response to transfected dsDNA (Fig. 1C). The levels of secreted IFN-β protein in culture supernatants from PTx pre-treated and cGAMP-stimulated cells (as in Fig. 1A) were quantified by ELISA and found to be congruent with *Ifnb1* mRNA (Fig. 1D). While we did not observe increases in STING-dependent cytokine mRNA (*Ifnb1* and *Cxcl10*) in response to PTx treatment alone at either 6 or 16 hrs, we additionally assayed for the ability of PTx to directly induce classical MyD88-dependent (e.g., COX2) and TRIF-dependent (e.g., viperin) genes using western blot. BMDMs were treated with TLR4 ligand *E. coli* LPS (10 ng/mL), STING ligand cGAMP (10 µg/mL), or purified PTx (250 ng/mL) for 18 hrs. The ability of each ligand to induce type I interferon or NF-κB/MAPK-dependent gene products was assayed by blotting for the accumulation of the IFN-dependent gene product viperin or the classical NF-κB/MAPK-dependent gene product COX2 (Fig. 1E). In line with previous literature, LPS induced both viperin and COX2 while cGAMP only induced viperin. PTx treatment, however, induced neither viperin nor COX2, arguing that in BMDMs, PTx alone is likely not directly activating a classical innate inflammatory response. We assayed the impact of PTx treatment on cGAMP-dependent IFN production by western blotting for viperin following cGAMP stimulation. Pre-incubation with PTx increased viperin protein expression in response to cGAMP (Fig. 1F; Fig. S1).

Collectively, these data indicate that PTx intoxication does not suppress cGAS/STING-dependent responses but rather significantly potentiates STING responses to cytosolic dsDNA or extracellular cGAMP.

**Fig 2:**
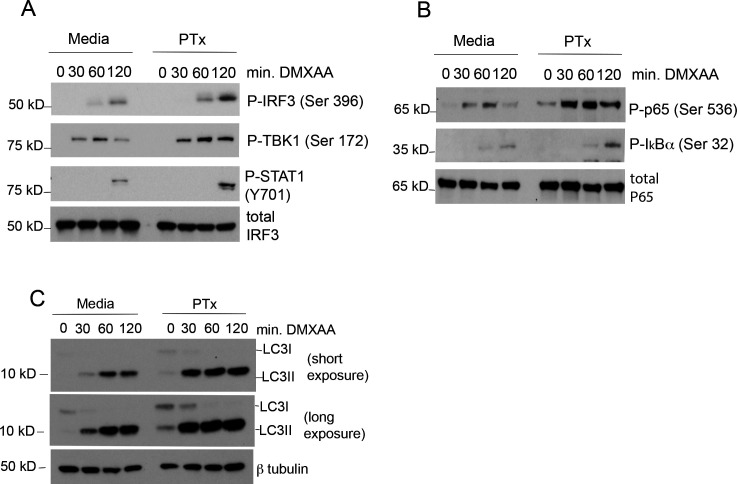
Pertussis toxin intoxication increases STING-dependent innate signaling. (**A–C**) BMDMs were cultured in media alone or intoxicated overnight (18 hrs) with 250 ng/mL of purified pertussis toxin. The following morning, media containing toxin were removed and replaced with fresh culture media. BMDMs were stimulated by the addition of STING ligand DMXAA for indicated times. Whole-cell lysates were harvested and analyzed by immunoblot using antibodies directed against the indicated protein targets. Representative of *n* = 5.

To begin investigating the mechanism(s) by which PTx enhances STING-dependent transcriptional responses, we examined the conserved innate signal transduction pathways driven by STING. As extracellular cGAMP must first be taken up by specific importers [e.g., SLC19A1 (28)], the kinetics of STING activation by exogenous cGAMP are somewhat variable between experiments, which complicates signal transduction studies. To alleviate this issue, STING activation in BMDMs was triggered by the use of the well-characterized, synthetic, small-molecule STING agonist DMXAA. DMXAA is cell-permeable, permitting it to drive robust STING activation with more defined kinetics in murine macrophages (29 – 31) than extracellular cGAMP. STING-mediated activation of the IRF3 transcription factor, which regulates the IFN-β promoter, was increased as a result of PTx treatment (Fig. 2A). STING-mediated activation of the NF-κB transcription factor pathway was also enhanced following exposure of BMDMs to PTx (Fig. 2B). In addition to the above pathways regulating transcription, STING drives a parallel pathway leading to the activation of an autophagic response (12). To ascertain whether exposure to PTx enhances STING-dependent autophagic signals, we examined the accumulation of the lipidated form of the autophagy-associated protein LC3 (referred to as LC3II) relative to the unlipidated LC3I. Intoxication by PTx also led to an enhanced DMXAA-dependent accumulation of LC3II relative to LC3I (Fig. 2C) consistent with an increased triggering of autophagy (32). These data indicate that PTx increases each of the known signal transduction pathways downstream of STING activation.

As our data indicate that all of the signaling pathways downstream of STING are similarly amplified by PTx intoxication, this suggests that PTx is likely not acting on a single signaling intermediate but may be acting at the level of STING itself.

**Fig 3:**
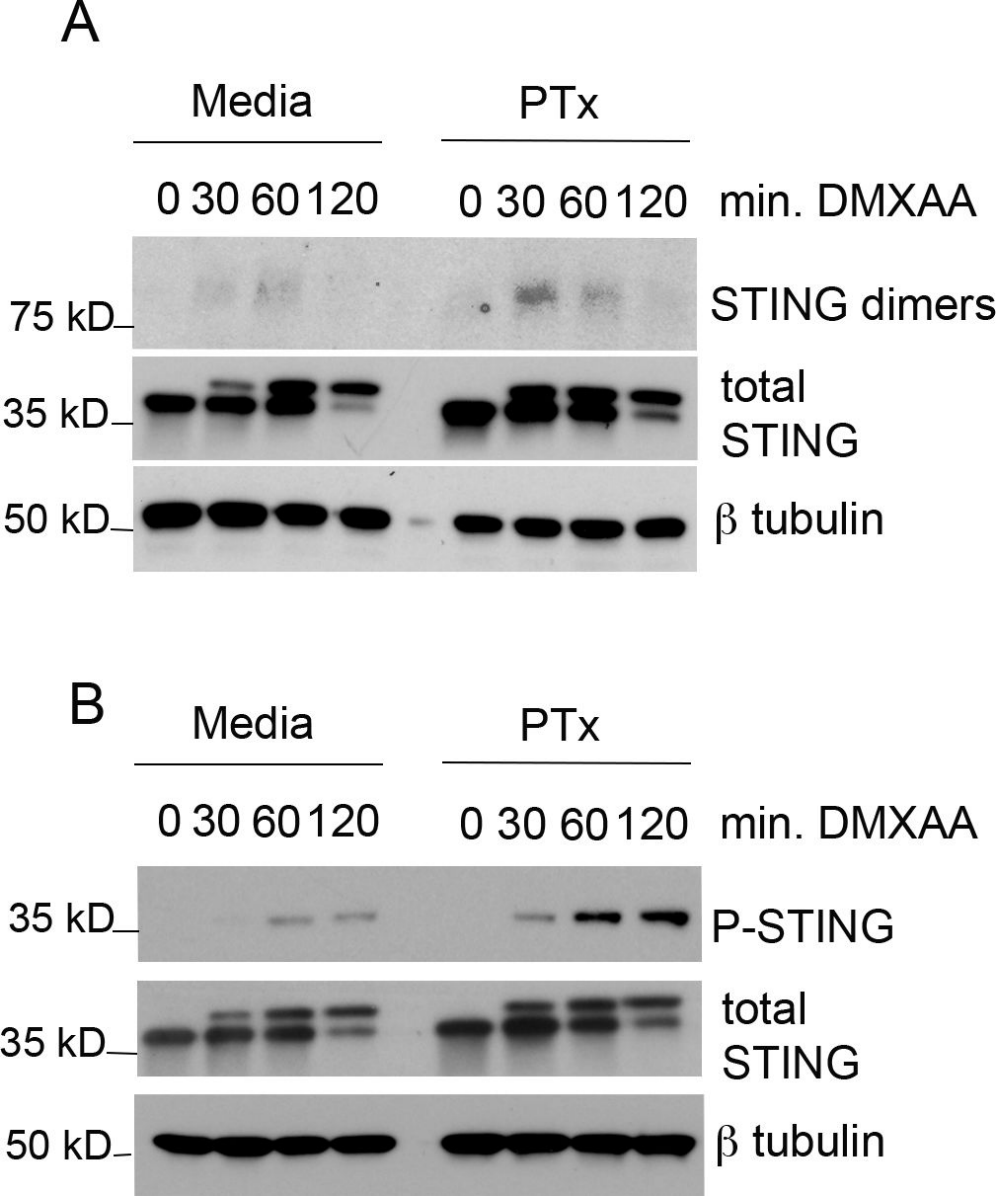
Pertussis toxin increases hallmarks of STING receptor activation by DMXAA. (**A**) BMDMs were cultured in media alone or intoxicated overnight with 250 ng/mL of purified pertussis toxin. The following morning, media containing toxin were removed and replaced with fresh culture media. BMDMs were stimulated by the addition of STING ligand DMXAA for indicated times. Whole-cell lysates were analyzed by immunoblot analysis under non-reducing conditions using the indicated antibodies. (**B**) BMDMs were stimulated as in (**A**), and whole-cell lysates were analyzed under standard reducing conditions using antibodies against the indicated protein targets. Representative of *n* = 3.

To determine whether PTx intoxication was acting at the level of the STING receptor, we examined early molecular events in STING receptor activation. Following ligand binding on the ER, STING monomers homodimerize, and these dimers are subsequently stabilized by the addition of disulfide linkage (8). STING dimers can, therefore, be visualized by western blot if samples are processed under non-reducing conditions. We observed an increase in STING dimer formation following DMXAA stimulation in PTx intoxicated BMDMs (Fig. 3A). Total STING protein expression levels were not appreciably altered by PTx (Fig. 3A and B). STING is well described to undergo a mobility shift in western blots following activation as a result of acquiring post-translational modifications. Following translocation from the ER, STING is phosphorylated by the kinase TBK-1 on a single residue (Ser365), an event that is required to license the STING scaffolding function and IRF3 activation (33). We assayed for the ligand-dependent phosphorylation of STING Ser365 and observed increased phosphorylation in macrophages treated with PTx (Fig. 3B). These results argue that PTx likely acts at or above the level of STING receptor activation.

**Fig 4:**
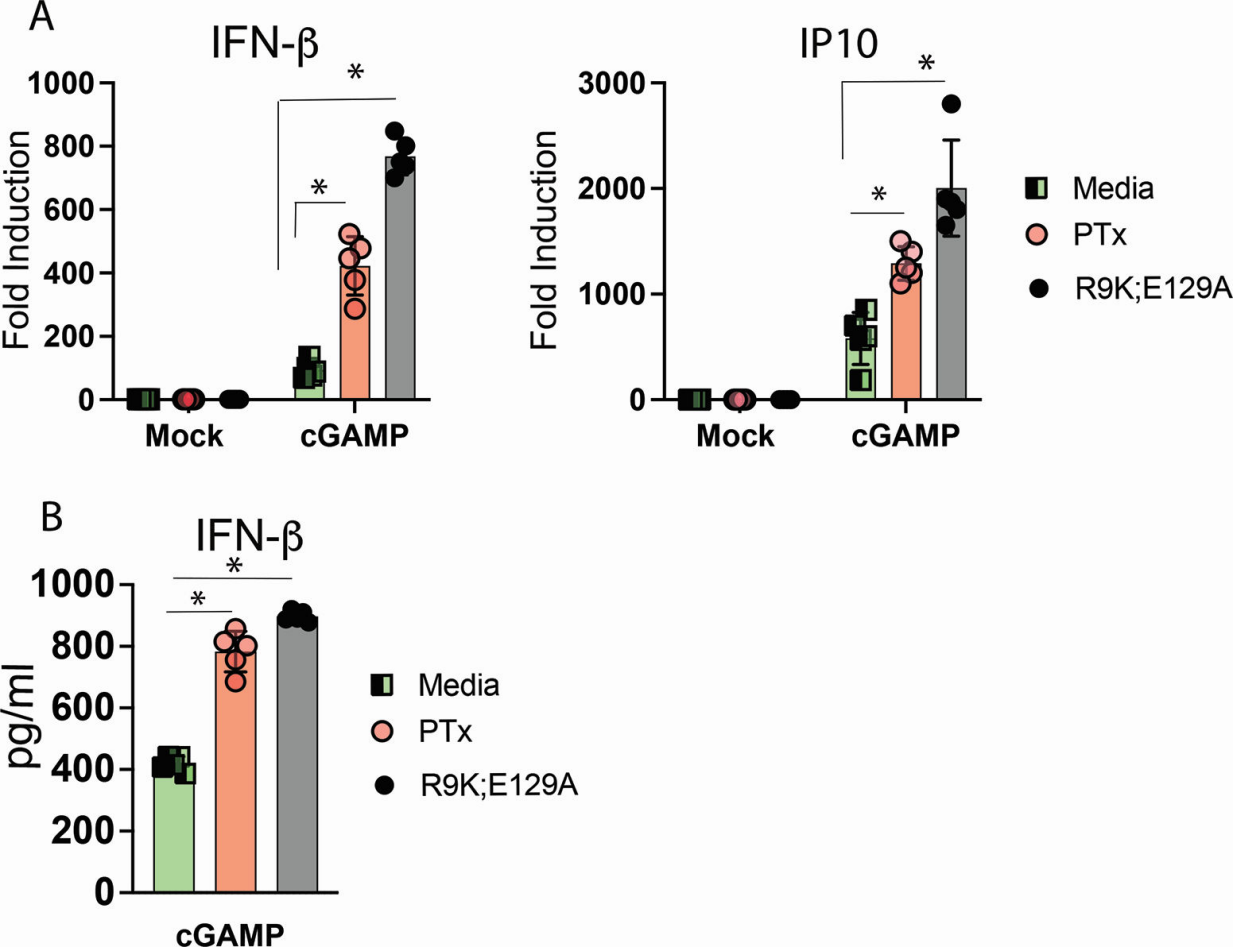
Pertussis toxin potentiates STING responses independent of ADP-ribosylating activity. (**A**) BMDMs were treated overnight with media alone, 250 ng/mL of WT, or 250 ng/mL R9K;E129A mutant purified pertussis toxin. The following morning, media containing toxin were removed and replaced with fresh culture media. BMDMs were stimulated by the addition of 10 µg/mL cGAMP to culture media for 4 hrs. Total RNA was harvested, and the expression of indicated genes was quantified by qRT-PCR. ^*^ *P* < 0.01. (**B**) Cell culture supernatants harvested at 4 hrs from macrophages treated in (**A**) were analyzed for the expression of IFN-β protein by ELISA. ^*^ *P* < 0.05.

We next asked whether the ADP-ribosylating activity of PTx was required to potentiate STING receptor activation. BMDMs were incubated overnight with equivalent doses of either the WT PTx or the R9K;E129A mutant of PTx. The R9K;E129A mutant has changes in two conserved catalytic amino acid residues and has been previously described to have a greater than 90% reduction in ADP-ribosylating activity *in vitro* (34, 35). Treatment of BMDMs with either WT or the R9K;E129A PTx resulted in enhanced cGAMP-dependent transcription of IFN-β and IP10 (Fig. 4A). Quantitation of secreted IFN-β protein in cell culture supernatants confirmed mRNA expression data (Fig. 4B). These data argue that the enzymatic activity of PTx is dispensable for its ability to enhance STING-dependent responses.

**Fig 5:**
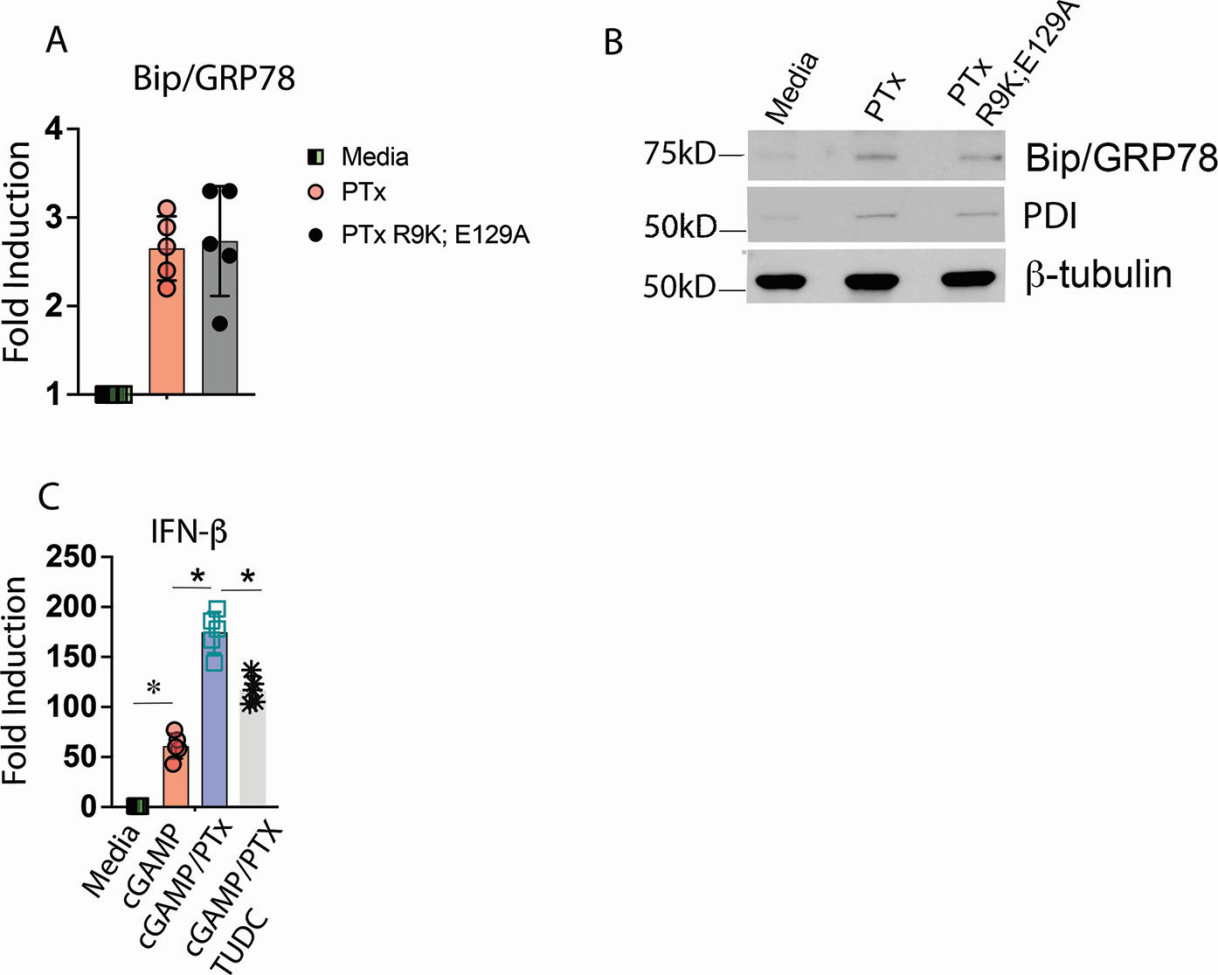
Pertussis toxin induces canonical markers of ER stress independent of catalytic activity, and ER stress contributes to the effect of PTx on STING. (**A**) BMDMs were treated overnight with media alone, 250 ng/mL of WT, or 250 ng/mL R9K;E129A mutant purified pertussis toxin. The following morning, media containing toxin were removed and replaced with fresh culture media for 4 hrs. Total RNA was then harvested, and the expression of Bip/GRP78 was quantified by qRT-PCR. (**B**) BMDMs were treated as in (**A**), and whole-cell lysates were harvested and analyzed by immunoblot for the indicated genes. Representative of *n* = 4 (**C**) BMDMs was cultured overnight with media alone or 250 ng/mL of WT PTx in the presence or absence of 100 µM TUDC. The following morning, media containing toxin was removed and replaced with fresh culture media. BMDMs were stimulated by the addition of 10 µg/mL cGAMP to culture media for 4 hrs. Total RNA was harvested, and the expression of indicated genes was quantified by qRT-PCR. ^*^ *P* < 0.01.

As ADP ribosylation is the only known enzymatic activity of PTx, we speculated that an additional aspect(s) of PTx interaction with host cell biology must be responsible for its effect on STING. Prior to ribosylating G_α_i complexes, PTx must gain entry to the cell by binding non-saturably to cell surface glycosylated molecules triggering its entry by endocytosis. Subsequent to entry into the cytosol, PTx traffics intracellularly by retrograde transport ultimately trafficking through the endoplasmic reticulum (25). In the ER, the catalytic A subunit of PTx separates from the B subunit pentamer, a step that is required for the A subunit to become enzymatically active. Once separated in the ER, the A subunit likely adopts an unfolded conformation enabling its subsequent ejection from the ER by the ER-Associated Decay (ERAD) transport pathway, whereby the active A subunit gains access to the cytosol enabling it to target G_α_i complexes (26, 36 – 38). PTx transit through the ER is of potential relevance as STING is an ER resident protein in the steady state, and STING activity can influence ER homeostasis. For example, STING activation is known to cause the induction of ER stress markers by poorly defined mechanisms (39 – 41). We, therefore, hypothesized that the entrance of the PTx holo-complexes into the ER and/or the adoption of an unfolded conformation by the catalytic A subunit triggering the ERAD pathway, perturbing ER homeostasis, is a manner sensed by STING. To test this idea, we treated BMDMs with WT or R9K;E129A PTx and assayed the mRNA expression of a canonical marker of ER stress, the chaperone Bip/GRP78. Bip is a master regulator of the ER stress response and in the steady state is associated with and inhibits multiple unfolded protein sensors (e.g., IRE, PERK, and ATF6). Following the accumulation of unfolded proteins in the ER, Bip has competed away from these unfolded protein response (UPR) sensors to associate with improperly folded proteins and prevent aggregation, which then allows for UPR sensor activation (42 – 44). Activation of the UPR sensors then triggers a transcriptional response including the rapid activation of the Bip gene, which increases the available pool of Bip protein. Overnight treatment with WT or R9K;E129A PTx induced an increase in steady-state Bip/GRP78 transcription, diagnostic of an ER stress response (Fig. 5A). Analysis of Bip/GRP78 protein levels by western blot indicated an increase in steady-state Bip protein consistent with our mRNA quantitation (Fig. 5B). Western blot analysis for the expression of a second canonical ER stress response protein, PDI, also was indicative of ER stress (Fig. 5B).

Complete pharmacologic elimination of ER stress is not technically feasible, and there is no animal model lacking all of the known ER stress sensors. It is, however, possible to pharmacologically buffer against a UPR-driven stress response. To do so, we used the “chemical chaperone” tauroursodeoxycholic acid (TUDCA). TUDCA is a widely used naturally occurring bile acid that can buffer against resulting ER stress in response to multiple triggers (45 – 47). We treated BMDMs simultaneously with TUDCA and PTx and measured subsequent transcriptional responses to cGAMP. TUDCA treatment significantly reduced the potentiating effect of PTx on STING consistent with a role for ER stress in the priming effect of PTx on STING (Fig. 5C).

**Fig 6:**
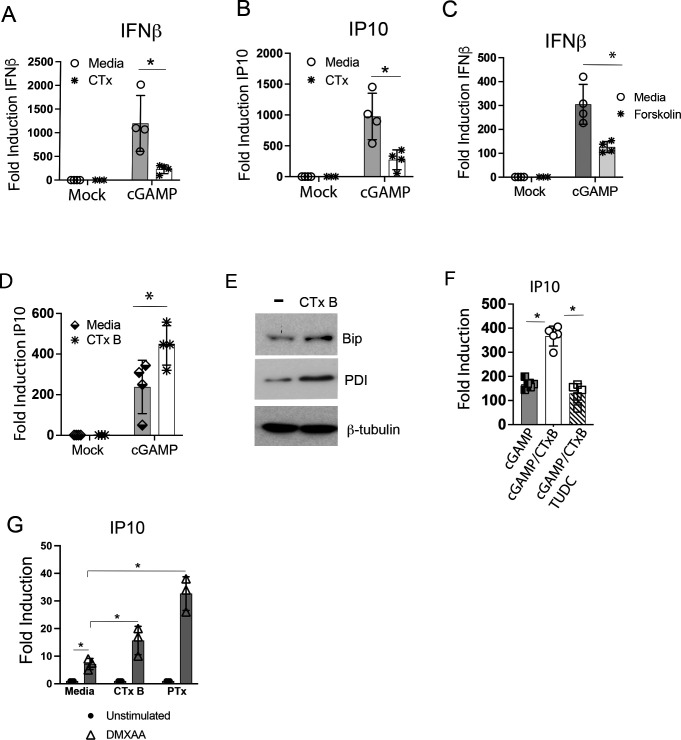
The catalytically inactive cholera toxin B subunit induces ER stress and primes STING responses. (A and B) BMDMs were cultured in media alone or intoxicated for 3 hrs with 10 µg of purified cholera toxin. BMDMs were subsequently stimulated by the addition of 10 µg/mL cGAMP to culture media for 4 hrs. Total RNA was harvested, and the expression of indicated genes was quantified by qRT-PCR. ^*^ *P* ≤ 0.05. (C) BMDMs were pre-treated with Forskolin for 3 hrs prior to stimulation with cGAMP for 4 hrs. Representative of *n* = 3. ^*^ *P* ≤ 0.05. (D) BMDMs were pre-treated with purified CTx B overnight prior to stimulation with 10 µg of cGAMP for 3 hrs. ^*^ *P* = 0.003. (E) BMDMs were treated with CTx B overnight as in (D). Whole-cell lysates were harvested and analyzed by immunoblot using antibodies directed against the indicated protein targets. Representative of *n* = 3. (F) BMDMs were cultured overnight with media alone or 20 ng of CTx B in the presence or absence of 100 µM TUDC. The following morning, media containing toxin were removed and replaced with fresh culture media. BMDMs were stimulated by the addition of 10 µg/mL cGAMP to culture media for 4 hrs. Total RNA was harvested, and the expression of indicated genes was quantified by qRT-PCR. ^*^ *P* < 0.05. *N* = 3. (G) Immortalized murine intestinal epithelial cells were treated overnight with media alone, 20 ng/mL CTx B, or 200 ng/mL PTx followed by stimulation with DMXAA for 4 hrs.

A second well-described ADP-ribosylating bacterial toxin, the CTx produced by *Vibrio cholerae,* shares the AB_5_ structural organization with PTx and also transits intracellularly through the ER in order to activate its catalytic subunits (48, 49). If an undefined perturbation of ER homeostasis by PTx was a necessary component to license enhanced STING activity, we hypothesized that the enhancement of STING responses may be a phenotype shared by PTx and CTx. To determine whether CTx similarly also regulates STING, we obtained purified CTx and treated BMDMs with CTx prior to stimulation with extracellular cGAMP. As we observed that overnight (16–20 hrs) intoxication by CTx resulted in significant cytotoxicity in BMDMs (data not shown), a 3-hr pre-incubation was used in these experiments. Unlike PTx, WT CTx intoxication for 3 hrs inhibited subsequent cGAMP-induced, STING-mediated transcription of both *Ifnb1* and *Cxcl10* (Fig. 6A and B). CTx inhibition of *Ifnb1* and *Cxcl10* correlated with reduced NF-κB activation by western blot in response to cGAMP (data not shown).

The most well-described molecular mechanism by which PTx and CTx enzymatic action contributes to the dysregulation of cellular homeostasis is via increasing the activity of the host cell adenylate cyclase (AC) enzyme activity. However, due to the distinct mechanism by which CTx drives adenylate cyclase, CTx intoxication is a far more potent activator of AC compared to PTx, and this effect underlies the extreme toxicity of CTx (50). It was, therefore, possible that the catalytic activity of WT CTx might be affecting STING-induced transcriptional responses through a high-magnitude increase in AC activity thereby masking ER-dependent effects. To determine whether enforcing high-level AC activation could inhibit STING, we employed a second pharmacologic approach to more specifically increase AC activity. Treating mammalian cells with the cell-permeable small-molecule Forskolin is known to directly activate AC enzymatic activity in a constitutive manner (51, 52). Similar to WT CTx intoxication, pre-treatment with Forskolin prior to stimulation with cGAMP reduced STING-dependent transcriptional responses (Fig. 6C), consistent with a role for supra-physiologic AC activity levels in restricting STING. The catalytically inactive CTx B pentamer alone is capable of binding to and entering mammalian cells in the absence of the A subunit but does not cause the cytotoxicity associated with the holotoxin (53). Despite lacking enzyme activity, CTx B has been demonstrated to have adjuvant activity *in vivo* although the relevant molecular interactions are undefined (54). We hypothesized that in the absence of the cytotoxic A subunit, CTx B trafficking to the ER may be sufficient to license an elevated STING response. To test this, BMDM cells were treated for 18 hrs with 20 ng/mL purified CTx B subunit (CTx B was generated by recombinant expression in mammalian HEK293 cells to eliminate any possible contaminating CTx A subunit), prior to stimulation with exogenous cGAMP. CTx B was capable of enhancing ligand-dependent STING transcriptional responses, albeit to a lesser extent than observed for PTx (Fig. 6D). To determine whether CTx B was also elevating markers of ER stress, we assayed Bip and PDI levels by western blot in media or CTx B-treated RAW264.7 cells. CTx B produced a similar elevation in both stress markers (Fig. 6E). Simultaneous treatment of BMDMs with CTx B (20 ng/mL) and the ER stress-reducing chemical chaperone TUDCA could significantly blunt elevated STING responses (Fig. 6F). To determine whether ER-transiting AB_5_ toxins could enhance STING responses in epithelial cells in addition to macrophages, we treated an immortalized murine intestinal epithelial cell line (gift of Dana Philpott) with PTx (200 ng/mL) or CTx B (20 ng/mL) for 18 hrs followed by STING activation with DMXAA. Both PTx and CTx B enhanced STING transcriptional responses in the epithelial background (Fig. 6G).

These data argue that both CTx and PTx can activate a similar STING priming pathway but that CTx enzymatic activity likely drives toxic effects masking the impact of the ER pathway.

## DISCUSSION

The cGAS/STING two-component sensor system is well described as a driver of type I interferons in response to the canonical microbial ligands for cGAS (dsDNA) or STING (cyclic nucleotides, e.g., c-GAMP). We have found that STING receptor activation and downstream transcriptional and autophagic pathways are markedly enhanced in murine macrophages treated with the pertussis toxin from *Bordetella pertussis* or cholera toxin B from *Vibrio cholerae* in response to these canonical ligands. Both of these toxins share the canonical AB_5_ structural architecture, and both must transit through the ER, where STING is resident, to activate their enzymatic activity. PTx or CTx B alone do not drive STING activation, as evidenced by the absence of transcriptional responses, but rather potentiate STING responses to a secondary ligand stimulus. This manner of molecular priming effect has not been described for STING previously. Notably, the action of PTx on STING does not require the ADP-ribosylating catalytic activity of PTx, which is required for full virulence of *B. pertussis* in *in vivo* models (17, 18). STING activation and signaling are known to be targeted for suppression by effectors from multiple bacterial pathogens including Ipa J from *Shigella*, CpoS from *Chlamydia*, and the phosphodiesterase CdnP from *Mycobacterium tuberculosis* (9, 55, 56). However, we have been unable to identify a prior instance in the literature of a reciprocal relationship in which STING activation is elevated by the impact of bacterial effectors.

Our findings may have relevance to an understanding of the reported action of PTx and CTx B as adjuvants in autoimmune and other disease models. *In vivo* PTx is routinely administered as an amplifying adjuvant to initiate disease in models of autoimmune uveitis and similarly is administered along with Freund’s adjuvant to drive disease in models of experimental autoimmune encephalitis (57, 58). The relevant molecular mechanisms behind the activity of PTx in these autoimmune models are poorly defined, but PTx administration is associated with increased antigen presenting cell (APC) activation and elevated innate cytokine production leading to enhanced T-dependent responses (59, 60). While the inhibition of G_α_i-coupled GPCR function by WT PTx almost certainly contributes to these effects on autoimmunity, enhanced activation of STING in response to self dsDNA released from dead and/or dying cells may also be a contributory mechanism. Similarly, CTx B has been used to adjuvant mucosal immune responses in several models (54, 61) although the molecular interactions required are undefined.

Our molecular data are congruent with the hypothesis that STING sensitivity to the PTx and CTx B toxin occurs, at least in part, through toxin-mediated perturbations in ER homeostasis. A model in which STING activation is potentiated and/or amplified by the effects of PTx and CTx B on ER homeostasis via the induction of aspects of an ER stress response is in agreement with current literature showing an intimate reciprocal relationship between STING and ER stress whereby STING activation leads to the induction of ER stress responses (39, 40, 62 – 65).

On an intermolecular basis, how PTx and CTx B induce ER stress requires a more granular study. It is clear that both toxins enter the ER after retrograde trafficking from the cell surface and then may trigger ER proteo-stress. For example, multiple copies of the toxin holo-complex entering the ER simultaneously may be sensed as misfolded protein aggregates triggering a UPR leading to Bip transcription. Additionally, the current model of PTX molecular activation holds that once in the ER, the S1 catalytic subunit of PTx dissociates from the pentameric binding “B” subunit and adopts an unfolded conformation, which triggers the ERAD protein disposal pathway leading to the ejection of S1 from the ER into the cytoplasm where it inactivates G protein complexes. Activation of the ERAD pathway by S1 may itself trigger the ER stress response. How exactly an ER stress response once triggered by PTx or CTx B can enhance ligand-dependent STING activation at the receptor level is unclear, but an important area of future research is the potential of toxin-associated ER stress to contribute to alterations in innate immune cellular functions as has recently been shown for *Streptococcus aureus (66*).

*In vivo*, PTx and CTx catalytic activities are required for the full pathogenesis of *B. pertussis* and *V. cholerae* and the complete loss of toxin expression affects many aspects of *in vivo* bacterial growth and host response (18, 67). It is not clear at present what impact the potentiation of STING might have on the systemic inflammatory response during infection as an *in vivo* relationship between *B. pertussis, V. cholerae,* and STING has not been reported in the literature. However, the *in vivo* production of type I/III IFNs is known to promote *B. pertussis*-driven pathology, a relationship shown for other Gram-negative pathogens as well (68, 69).

Other bacterial toxins are also known to enter the ER and induce varying degrees of ER stress by assorted mechanisms including VceC from *Brucella abortus* (70, 71), VacA toxin from *Helicobacter pylori* (72), and subtilase toxin of *E. coli* (73). It is conceivable that these additional toxins may share the ability to potentiate STING responses.

## Data Availability

The authors assert that materials and data that are reasonably requested by others will be available from a publicly accessible collection or will be made available in a timely fashion, at reasonable cost, and in limited quantities to members of the scientific community for non-commercial purposes.

## SUPPLEMENTAL MATERIAL

Fig. S1 iai.00300-24-s0001.tif Densitometry of Western blot bands.

ASM does not own the copyrights to Supplemental Material that may be linked to, or accessed through, an article. The authors have granted ASM a non-exclusive, world-wide license to publish the Supplemental Material files. Please contact the corresponding author directly for reuse.
