# Supplementary figures and images for: ER-transiting bacterial toxins amplify STING innate immune responses and elicit ER stress
Source: Infect Immun. 2024 Jul 26;92(8):e00300-24. doi: 10.1128/iai.00300-24 (PMC11321001; doi:10.1128/iai.00300-24)

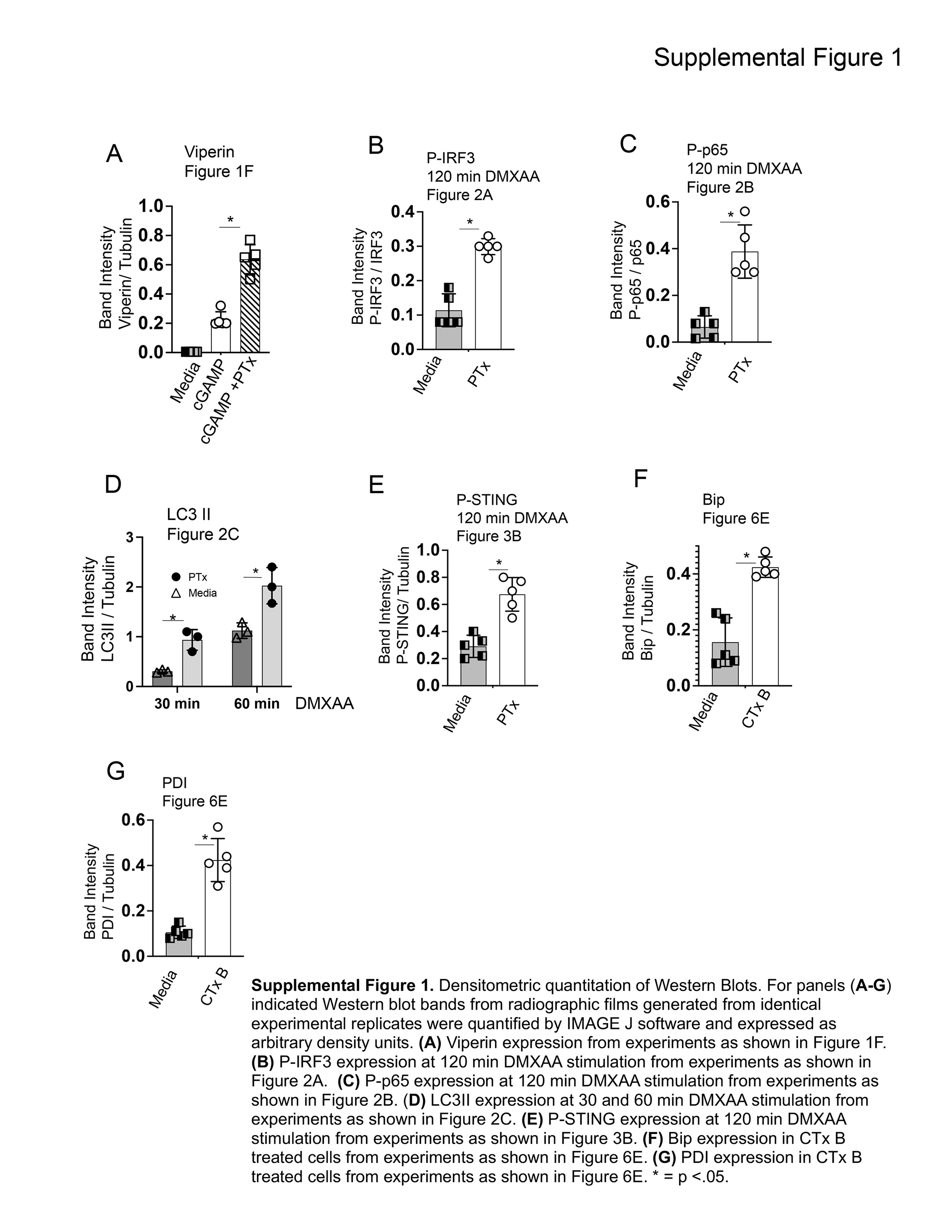

Supplement: Fig. S1 — Densitometry of Western blot bands. [file iai.00300-24-s0001.tif]
